# Supplementary material for: Lazertinib versus Platinum-Based Chemotherapy with Epidermal Growth Factor Receptor (EGFR)-Positive Non-Small-Cell Lung Cancer after Failing EGFR-Tyrosine Kinase Inhibitor: A Real-World External Comparator Study
Source: Cancers (Basel). 2024 Jun 7;16(12):2169. doi: 10.3390/cancers16122169 (PMC11202219; doi:10.3390/cancers16122169)
Supplement: Supplementary file 1 [file cancers-16-02169-s001.zip › cancers-3030944-supplementary.pdf]

Supplementary Materials

Table S1. Summary of the propensity score model.

	**Mainmodel**	**Alternative model 1**	**Alternative model 2**
Age	O	O	O
Sex	O	O	O
History of smoking	O	O	O
ECOG performance status	O	O	O
Brain metastasis	O	O	O
Previous lines of EGFR-TKI treatment	O		O
Type of previous EGFR-TKI treatment		O	O
Time from last EGFR-TKI treatment	O	O	O
Duration of previous EGFR-TKI treatment	O	O	O
C-statistics	0.773	0.803	0.831

ECOG, Eastern Cooperative Oncology Group; EGFR-TKI, epidermal growth factor receptor-tyrosine kinase inhibitor.

The 'O' indicates whether an individual confounder was included in the outcome model

Table S2. Results of progression-free survival, overall survival, and time to treatment discontinuation before and after propensity score matching.

	Before PS matching			After PS matching		
	**Lazertinib (*n* = 200)**	**External comparator (*n* = 334)**	***p*-value**	**Lazertinib (*n* = 156)**	**External comparator (*n* = 156)**	***p*-value**
**Progression-free Survival**						
No. of patients, n (%)	127 (63.5)	311 (93.1)		101 (64.7)	146 (93.6)	
Progression	119 (59.5)	296 (88.6)		96 (61.5)	137 (87.8)	
Death	8 (4.0)	15 (4.5)		5 (3.2)	9 (5.8)	
Incidence rate per 100 PYs	66.39	171.95		69.17	176.04	
Time to PFS, median (95% CI)	12.16 (9.72-13.63)	5.03 (4.80-5.60)	<0.001	10.97 (8.77-12.52)	5.10 (4.67-6.07)	<0.001
Crude HR (95% CI)	0.38 (0.31-0.47)	1.00 (Reference)	<0.001	0.39 (0.30-0.51)	1.00 (Reference)	<0.001
Adjusted HR (95% CI)[a]	0.40 (0.31-0.53)	1.00 (Reference)	<0.001	0.40 (0.29-0.55)	1.00 (Reference)	<0.001
**Overall Survival**						
No. of patients, n (%)	50 (25.0)	197 (59.0)		39 (25.0)	94 (60.3)	
Incidence rate per 100 PYs	18.27	41.21		18.26	41.98	
Time to OS, median (95% CI)	33.84 (30.13-NE)	20.00 (17.03-23.90)	<0.001	32.23 (29.08-NE)	18.73 (14.40-24.87)	<0.001
Crude HR (95% CI)	0.45 (0.33-0.61)	1.00 (Reference)	<0.001	0.44 (0.30-0.64)	1.00 (Reference)	<0.001
Adjusted HR (95% CI)[a]	0.48 (0.33-0.70)	1.00 (Reference)	<0.001	0.45 (0.29-0.69)	1.00 (Reference)	<0.001
**Time to Treatment Discontinuation**						
No. of patients, n (%)	151 (75.5)	266 (79.6)		120 (76.9)	123 (78.8)	
Incidence rate per 100 PYs	69.32	133.79		71.37	134.46	
Time to TTD, median (95% CI)	12.32 (10.41-13.83)	6.53 (5.73-6.97)	<0.001	11.66 (9.10-13.83)	6.73 (5.50-7.10)	<0.001
Crude HR (95% CI)	0.48 (0.39-0.59)	1.00 (Reference)	<0.001	0.50 (0.39-0.65)	1.00 (Reference)	<0.001
Adjusted HR (95% CI)[a]	0.54 (0.41-0.71)	1.00 (Reference)	<0.001	0.54 (0.39-0.75)	1.00 (Reference)	<0.001

PS, propensity score; PY, person-year; CI, confidence interval; PFS, progression-free survival; HR, hazard ratio; OS, overall survival; NE, not estimated; TTD, time to treatment discontinuation.

[a]Imbalanced potential confounders were adjusted as covariates.

Figure S1. Duration of progression-free survival before propensity score matching.

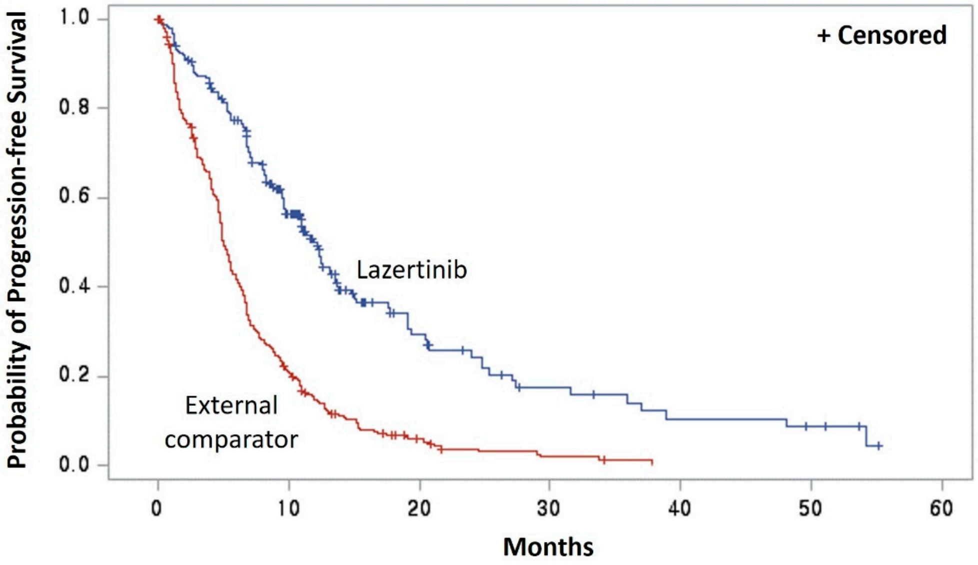

	No. of Patients	Median Progression-free Survival *mo (95% CI)*
Lazertinib	127/200	12.16 (9.72-13.63)
External comparator	311/334	5.03 (4.80-5.60)

Adjusted Hazard Ratio for Progression-free Survival before PSM
: 0.40 (95% CI, 0.31-0.53); p-value: <0.001

**No. at Risk**							
Lazertinib	200	93	25	11	6	4	0
External comparator	334	68	12	3	0	0	0

Abbreviations: PSM, propensity score matching**.**

Figure S2. Duration of overall survival before propensity score matching.

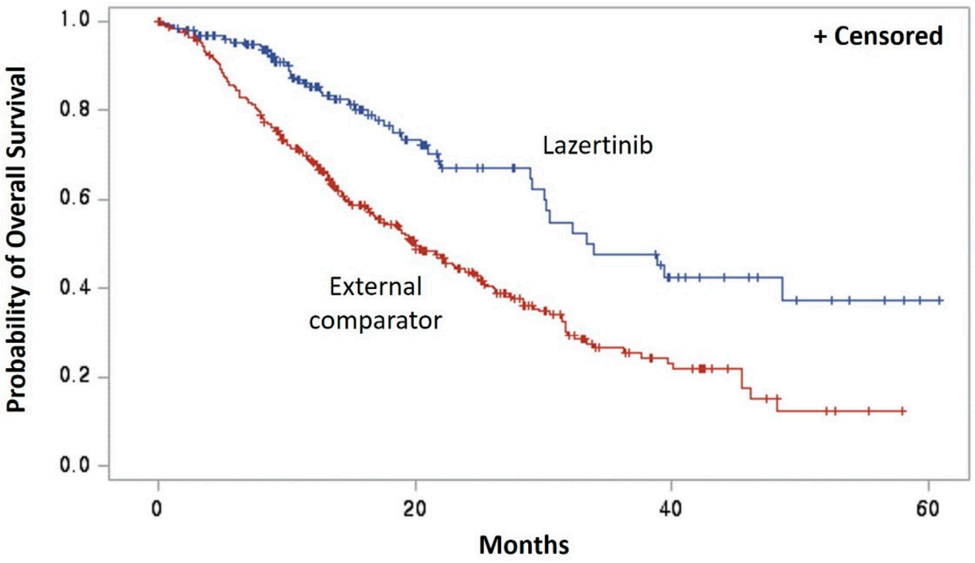

	No. of Patients	Median Overall Survival *mo (95% CI)*
Lazertinib	50/200	33.84 (30.13-NE)
External comparator	197/334	20.00 (17.03-23.90)

Adjusted Hazard Ratio for Overall Survival before PSM
: 0.48 (95% CI, 0.33-0.70); p-value < 0.001

**No. at Risk**				
Lazertinib	200	49	14	1
External comparator	334	117	18	0

Abbreviations: PSM, propensity score matching**.**

Figure S3. Duration of time to treatment discontinuation before propensity score matching.

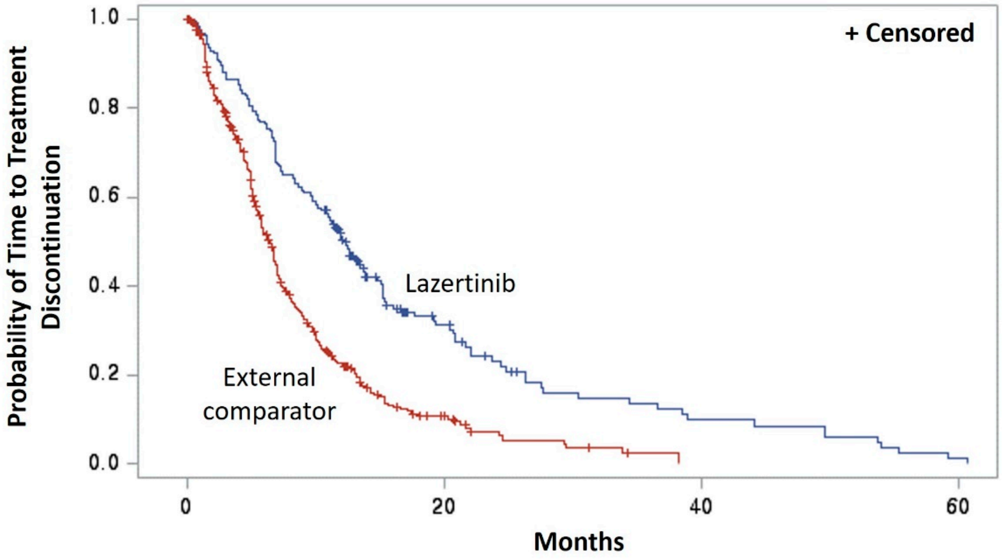

No. at Risk				
Lazertinib	200	32	8	1
External comparator	334	18	0	0

	No. of Patients	Median Time to Treatment Discontinuation *mo (95% CI)*
Lazertinib	151/200	12.32 (10.41-13.83)
External comparator	266/334	6.53 (5.73-6.97)

Adjusted Hazard Ratio for
Time to Treatment Discontinuation before PSM
: 0.54 (95% CI, 0.41-0.71); p-value: <0.001

Abbreviations: PSM, propensity score matching.
